# Outcomes of Single-Step Transepithelial Photorefractive Keratectomy Compared With Alcohol-Assisted Photorefractive Keratectomy Using Wave-Light EX500 Platform

**DOI:** 10.7759/cureus.36872

**Published:** 2023-03-29

**Authors:** Abrar Alhawsawi, Jumana Hariri, Mohanna Aljindan, Khalid Alburayk, Hammam A Alotaibi

**Affiliations:** 1 Department of Ophthalmology, Faculty of Medicine, University of Jeddah, Jeddah, SAU; 2 Department of Ophthalmology, Dhahran Eye Specialist Hospital, Dhahran, SAU; 3 Department of Ophthalmology, King Fahad University Hospital, Imam Abdulrahman Bin Faisal University, Khobar, SAU; 4 Research Center, Prince Sultan Military Medical City, Riyadh, SAU

**Keywords:** alcohol-assisted prk, moderate myopia, errors of refraction, refractive surgeries, trans prk

## Abstract

Purpose

To compare the visual outcome of transepithelial photorefractive keratectomy (PRK) against alcohol-assisted PRK in treating low-to-moderate myopia with or without astigmatism.

Setting

Dhahran Eye Specialist Hospital, Dhahran, Saudi Arabia.

Design

This is a retrospective study.

Methods

Forty eyes of 22 patients with myopia from -0.75 to -6.00 diopters (D) with or without astigmatism from 0 to -3D were included in this study. Preoperative and postoperative data of 20 eyes from 11 patients who underwent transepithelial PRK were compared with 20 eyes from 11 patients who underwent alcohol-assisted PRK were collected and analyzed. The uncorrected distance visual acuity (UDVA), corrected distance visual acuity (CDVA), manifest spherical equivalence (SE), manifest cylinder, vector analysis of astigmatism, and efficacy and safety indices were compared between the groups at a mean follow-up of one year postoperatively.

Results

Baseline characteristics were similar between groups, except the transepithelial PRK group had lower cylinder values than the alcohol-assisted PRK group by 0.69D. Regression analysis was used to control for the difference in the cylinder in all outcome parameters. Both groups had similar mean UDVA (p=0.73), CDVA (p=0.98), the proportion of eyes in either group achieved (20/20, 20/25, and 20/30) UDVA (p=0.72, 0.68 and 0.31 respectively) and percentage of eyes lost two lines of CDVA (p=1.0). There was no statistically significant difference between the two groups in regard to both efficacy and safety indices (p=0.55 and 0.67, respectively). Both groups had similar residual SE (p=0.72), the proportion of eyes within ±0.5D of SE (p=0.29), and residual refractive astigmatism (p=0.87). Both groups had similar difference vectors, surgically induced astigmatism, and correction index (p=0.82, 0.10, and 0.26, respectively). However, the transepithelial PRK group had lower target-induced astigmatism (*TIA; p*=0.01), higher magnitude of error (ME; p=0.05), and higher angle of error (AE; p=0.02) than the alcohol-assisted PRK group.

Conclusion

Transepithelial PRK had similar visual and refractive outcomes as alcohol-assisted PRK. This approach was considered as safe and effective as alcohol-assisted PRK in treating patients with low-to-moderate myopia with or without astigmatism.

## Introduction

Photorefractive keratectomy (PRK) is a well-known method to treat refractive errors [1]. Transepithelial PRK was first introduced in the 1990s [2], and several improvements have been developed since the technique’s introduction. In 1999, a two-step procedure was introduced using phototherapeutic keratectomy followed by PRK. In 2007, a single-step PRK was developed. A reverse single-step was introduced in 2012 and refined in 2016, and a smart pulse technology was included in 2017 [3-6]. The goal of PRK is to remove the corneal epithelium either mechanically, chemically, or via excimer laser and then ablate the stroma with the excimer laser. In single-step transepithelial PRK, the excimer laser removes the corneal epithelium and ablates the stroma in a single step [7]. Because the epithelium is removed by the laser, the corneal epithelial defect (CED) has regular, precisely defined edges and a smooth surface, resulting in faster healing time, less haze, and less pain postoperatively than other procedures [6,7]. Several studies compared visual, clinical, and refractive outcomes between different single-step transepithelial PRK techniques and other laser-assisted keratorefractive techniques, including alcohol-assisted PRK, mechanical PRK, laser in situ keratomileusis (LASIK), femtosecond-assisted LASIK, and laser epithelial keratomileusis. Most of these studies showed similar efficacy between transepithelial PRK and the other procedure [8-17]. Few studies showed better visual acuity (VA) and refractive results in the transepithelial PRK patients than patients receiving alcohol-assisted PRK and LASIK [5,18], with faster visual rehabilitation and less pain and haze for transepithelial PRK patients [13,14,18,19]. Other non-comparative studies showed a promising and acceptable outcome of single-step transepithelial PRK [4,20-23]. This study aims to compare transepithelial PRK against alcohol-assisted PRK for VA and manifest refraction in treating patients with low-to-moderate myopia with or without astigmatism.

## Materials and methods

This is a retrospective study of all patients who underwent transepithelial PRK and alcohol-assisted PRK at the Dhahran Eye Specialist Hospital between September 2019 and January 2020. The transepithelial PRK group was retrospectively chosen, then the alcohol-assisted group was chosen and matched for age, gender, and spherical equivalence (SE). All procedures were conducted by four experienced surgeons using the same excimer laser machine Wave-Light® EX500 (Alcon Laboratories, Fort Worth, TX, USA). The study was approved by Institutional Review Board Ethical Committee at Dharan Eye Specialist Hospital. The study includes patients aged 18 years or older with stable preoperative refraction for at least one year, low-to-moderate myopia from -0.75 to -6.00 diopters (D), astigmatism from 0.00 to -3D, and follow-up duration of at least six to 12 months. The study excluded patients with coexisting ocular pathology (e.g., keratoconus, keratitis, and dystrophies) or previous ocular surgery (e.g., add-on PRK, LASIK, and corneal transplant) and hyperopia. Patients were also excluded if they were pregnant or had coexisting systemic diseases such as diabetes or connective tissue disease. Preoperative and postoperative evaluations included distant VA (both uncorrected distance VA [UDVA] and corrected distance VA [CDVA]) using the Snellen chart, intraocular pressure using a pneumotonometer, manifest refraction, anterior segment examination, and dilated fundus examination using slit-lamp biomicroscopy and corneal tomography using a Pentacam (Oculus, Germany). Patients were asked to discontinue contact lens use at least three weeks before tomographic and subjective refraction evaluations. Patients provided informed consent before the surgical procedure. Following sterilization and draping of the eyes in the usual manner, topical anesthetic drops were applied, and an eyelid speculum was inserted. In the alcohol-assisted group, the epithelium was removed by marking the cornea by optical zone (OZ; 8.5 mm), applying the alcohol (ethanol 30%) for 30 seconds, then absorbing the alcohol with a small sponge. Then the area was thoroughly irrigated with a balanced saline solution (BSS), and the epithelium was removed using a cotton-tipped stick. In the transepithelial PRK group, the epithelium was removed using stream light software of WaveLight EX500 machine, taking out a fixed amount of epithelial thickness (50 to 55 microns) in all eyes. Both groups received stromal ablation using the standard wavefront optimized profile of WaveLight nomograms that were based on manifest refraction with an OZ of 6.5 mm for all eyes in both groups. A sponge soaked in topical mitomycin C 0.02% (Kyowa Hakko Co. Ltd., Japan) was applied based on the ablation depth. Finally, the eyes were irrigated again with BSS and protected by a soft bandage contact lens (Pure Vision, Bausch & Lomb, Rochester, NY) for one week. All treatments were targeted for emmetropia. Patients were instructed in postoperative consisting of moxifloxacin drops (Vigamox, Alcon Laboratories, Inc., Fort Worth, Texas, USA) four times per day for two weeks and prednisolone acetate 1% (pred forte; Allergan, Irvine, CA) four times per day, tapering over one month. Patients were evaluated one week, one month, three months, six months, and one year after the procedure.

The study’s primary outcomes were comparing UDVA, CDVA, SE deviation from the target, and manifest cylinder. Target-induced astigmatism (TIA) is the astigmatic change by magnitude and axis the surgery was intended to induce). Surgically induced astigmatism (SIA) is the amount and axis of astigmatic change the surgery actually induces. Correction index (CI) is the ratio of the SIA to TIA; CI > 1.0 indicates an overcorrection, and CI < 1.0 indicates an under correction. The magnitude of error (ME) is the arithmetic difference between the magnitude of the SIA and TIA. The angle of error (AE) is the angle described by the SIA and TIA. AE is positive if the axis of the SIA is counterclockwise to the axis of the TIA and negative if the axis of the SIA is clockwise to the axis of the TIA. The difference vector (DV) is the change that would enable the initial surgery to achieve the original target on the second attempt (DV is preferably 0). The efficacy index is the ratio of postoperative UDVA to preoperative CDVA in decimal notation. The safety index is the ratio of postoperative CDVA to preoperative CDVA in decimal notation. CDVA was converted to a logarithm of the minimum angle of resolution (LogMar) for analysis. Statistical analysis was performed with IBM SPSS Statistics for Windows, Version 22.0. (IBM Corp., Armonk, NY, USA). All figures were constructed with Microsoft Excel (2019, Microsoft Corp., Redmond, WA, USA). The normality of the data was assessed by the Shapiro-Wilk test. Normally distributed data were compared with the unpaired t-test. Non-normally distributed data were compared with the Mann-Whitney U test. Categorial data were compared with the Chi-squared test. P < 0.05 was considered statistically significant.

## Results

A total of 40 eyes from 22 patients (6 men and 16 women) were included, 20 eyes (11 patients) in the transepithelial PRK group, and 20 eyes (11 patients) in the alcohol-assisted PRK group. Table 1 presents the baseline characteristics of eyes in both groups, which were similar except for cylinder: eyes in the transepithelial PRK group had lower values than those in the alcohol-assisted PRK group by 0.69D.

**Table 1:** Baseline characteristics of the eyes in transepithelial PRK and alcohol-assisted PRK. CDVA: Corrected distance visual acuity; D: Diopter; F: Female; K: Keratometry reading; LogMar: Logarithm of minimum angle of resolution; M: Male; PRK: Photorefractive keratectomy; SE: Spherical equivalent; UDVA: Uncorrected distance visual acuity.

	Transepithelial PRK (n=20)		Alcohol-assisted PRK (n=20)		P-value
	Mean ± SD	Range	Mean ± SD	Range	
Age (years)	29.1 ± 5.9	19.0 to 39.0	28.6 ± 6.5	18.0 to 36.0	0.78
M: F Ratio	3:8		3:8		1.0
CDVA (logMAR)	0.01 ± 0.02	0 to 0.10	0.01 ± 0.03	0 to 0.15	0.97
SE (D)	-3.55 ± 1.71	-6.38 to -1.00	-4.01 ± 1.31	-5.88 to -1.75	0.35
Sphere (D)	-3.33 ± 1.72	-6.00 to -1.00	-3.44 ± 1.22	-5.75 to -0.75	0.81
Cylinder (D)	0.45 ± 0.45	1.50 to 0	1.14 ± 0.94	3.00 to 0	0.01
K 1 flat (D)	42.45 ± 1.88	38.97 to 45.86	42.68 ± 0.97	41.46 to 44.44	0.64
K2 steep (D)	43.46 ± 1.81	40.04 to 46.75	44.37 ± 1.31	42.29 to 46.87	0.08
Follow-up (month)	13.18 ± 3.08	5.22 to 16.66	13.85 ± 1.70	11.40 to 16.43	0.94

Table 2 summarizes the intraoperative data. Both groups had similar intraoperative parameters except for transition zone (TZ), ablation zone (AZ), central corneal thickness (CCT), and ablation time. The transepithelial PRK group had a smaller mean TZ by 0.33 mm, smaller AZ by 0.67 mm, and smaller CCT by 25 microns than the alcohol-assisted PRK group. The transepithelial group had a longer mean ablation time (by 37 seconds) than the alcohol-assisted PRK (P < 0.001).

**Table 2:** Intraoperative data. AZ: Ablation zone; CCT: Central corneal thickness; OZ: Optical zone; PRK: Photorefractive keratectomy; TZ: Transitional zone.

	Transepithelial PRK (n=20)		Alcohol-assisted PRK (n=20)		
Parameter	Mean ± SD	Range	Mean ± SD	Range	P-value
OZ (mm)	6.51 ± 0.02	6.50 to 6.60	6.50 ± 0	6.50 to 6.50	0.32
TZ (mm)	0.87 ± 0.48	0.30 to1.25	1.20 ± 0.21	0.30 to 1.25	0.01
AZ (mm)	8.24 ± 0.95	7.10 to 9.00	8.91 ± 0.42	7.10 to 9.00	0.01
CCT (µm)	525 ± 26	488 to 574	550 ± 35	460 to 613	0.01
Depth of ablation (µm)	72 ± 70	16 to 353	68 ± 21	31 to 101	0.25
Residual thickness (µm)	399 ± 82	90 to 496	432 ± 43	329 to 499	0.08
Laser exposure time (second)	50 ± 19	30 to 118	13 ± 10	4 to 53	<0.001

Visual acuity

At a mean follow-up of approximately one year after the procedure, the differences in mean UDVA (P = 0.73) and CDVA (P = 0.98) were not statistically significant between the two groups (Table 3). The difference in percentages of eyes in both groups that achieved 20/20 (P = 0.72), 20/25 (P = 0.68), and 20/30 UDVA (P = 0.31) were not statistically significant (Table 3). Also, the difference between the groups in eyes that lost two lines of CDVA was not statistically significant (P = 1.0).

**Table 3:** Postoperative refraction and visual outcome at one year follow-up for transepithelial PRK and alcohol-assisted PRK. CDVA: Corrected distance visual acuity; D: Diopter; K; Keratometry reading; logMar: Logarithm of minimum angle of resolution; PRK: Photorefractive keratectomy; SE: Spherical equivalent; UDVA: Uncorrected distance visual acuity.

Parameter	Transepithelial PRK (n=20)	Alcohol-Assisted PRK (n=20)	P-value
UDVA (logMAR), mean ± SD (range)	0.04 ± 0.07 (0 to 0.18)	0.04±0.08 (0 to 0.30)	0.73
CDVA (logMAR), mean ± SD (range)	0.01 ± 0.04 (0 to 0.18)	0.02±0.05 (0 to 0.15)	0.98
Eyes at 20/20 UDVA, n (%)	14 (70%)	15 (75%)	0.72
Eyes at 20/25 UDVA, n (%)	16 (80%)	17 (85%)	0.68
Eyes at 20/30 UDVA, n (%)	20 (100%)	19 (95%)	0.31
Eyes lost 2 or more lines of CDVA, n (%)	1 (5%)	1 (5%)	1.0
SE in D, mean ± SD (range)	-0.01 ± 0.32 (-0.50 to 0.88)	-0.09 ± 0.58 (-2.00 to 0.63)	0.72
Eyes within ±0.5 D from the target SE, n (%)	19 (95%)	17 (85%)	0.29
Change in SE in D, mean ± SD (range)	-3.54 ± 1.83D (-6.88 to -1.00D)	-3.92 ± 1.34D (-6.25 to -1.50D)	0.46
Sphere in D, mean ± SD (range)	0.11 ± 0.32 (-0.25 to 1.00)	0.04 ± 0.51 (-1.50 to 1.00)	0.82
Cylinder in D, mean ± SD (range)	0.24 ± 0.26 (0 to 0.75)	0.25 ± 33 (0 to 1.00)	0.87
Eyes with cylinder between (0-0.5D), n (%)	19 (95%)	18 (90%)	0.55
K1 flat in D, mean ± SD (range)	38.96 ± 1.50 (35.10 to 41.80)	39.25 ± 1.35 (37.90 to 42.30)	0.75
K2 steep in D, mean ± SD (range)	39.62 ± 1.74 (35.60 to 42.20)	40.09 ± 1.42 (38.70 to 43.30)	0.39
Pachymetry in µm, mean ± SD (range)	474 ± 58 µm (358 to 551 µm)	499 ± 50 µm (392 to 556 µm)	0.08
Thinnest location in µm, mean ± SD (range)	464 ± 53 µm (357 to 549 µm)	494 ± 50 µm (388 to 554 µm)	0.04

Table 4 presents efficacy and safety indices. There were no statistically significant differences in efficacy (P = 0.55) or safety indices (P = 0.67) between the groups.

**Table 4:** Efficacy and safety indices for transepithelial PRK and alcohol-assisted PRK. PRK: Photorefractive keratectomy.

	Transepithelial PRK	Alcohol-assisted PRK	
Index	Mean ± SD (range)	Mean ± SD (range)	P-value
Efficacy	0.93 ± 0.12 (0.66 to 1.00)	0.94 ± 0.14 (0.50 to 1.00)	0.55
Safety	0.99 ± 0.09 (0.66 to 1.15)	0.98 ± 0.07 (0.71 to 1.00)	0.67

**Figure 1:**
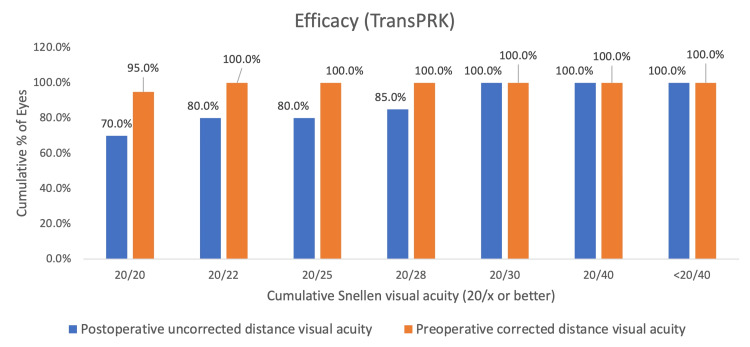
Efficacy of transepithelial PRK. PRK: Photorefractive keratectomy.

**Figure 2:**
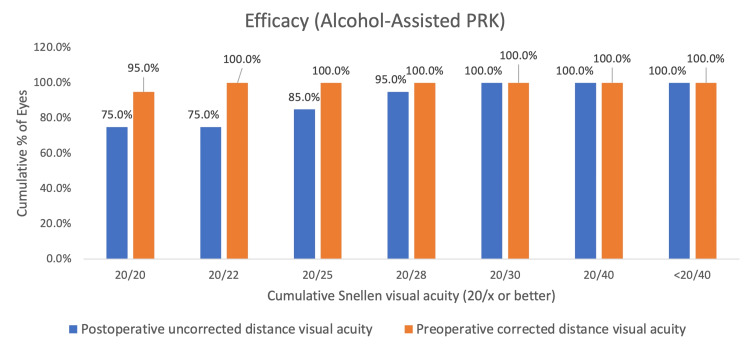
Efficacy of alcohol-assisted PRK. PRK: Photorefractive keratectomy.

**Figure 3:**
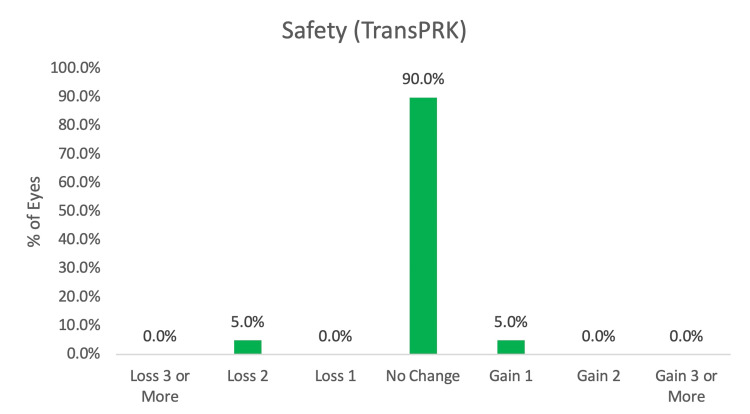
Safety of transepithelial PRK. PRK: Photorefractive keratectomy.

**Figure 4:**
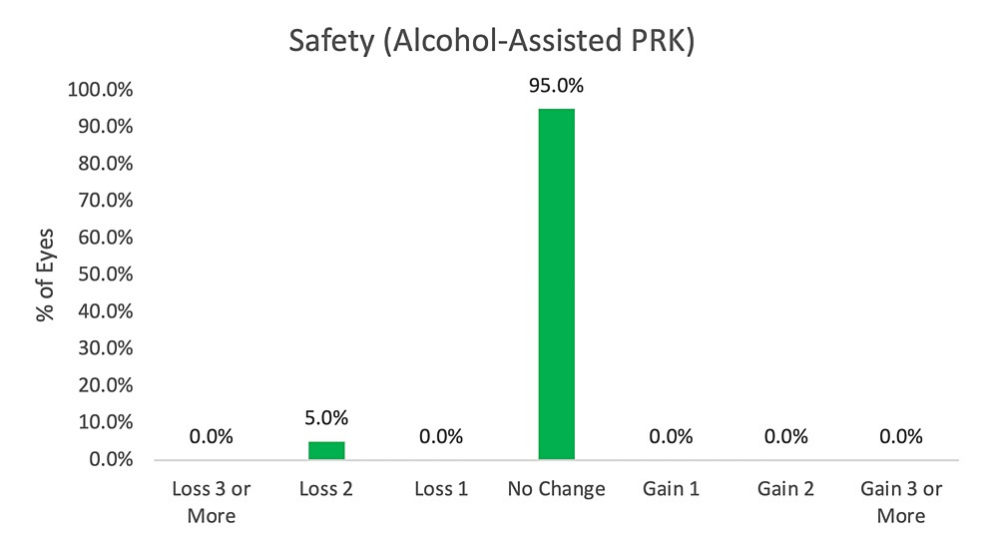
Safety of alcohol-assisted PRK. PRK: Photorefractive keratectomy.

Figures 1-4 show the efficacy and safety profiles for the transepithelial PRK and alcohol-assisted PRK groups. Regression analysis after controlling for cylinder shows that both groups have a similar efficacy (Rsquare = 0.063, P = 0.445) and safety indices (Rsquare = 0.011, P = 0.94).

Refractive results and accuracy

Spherical Equivalence (SE)

**Figure 5:**
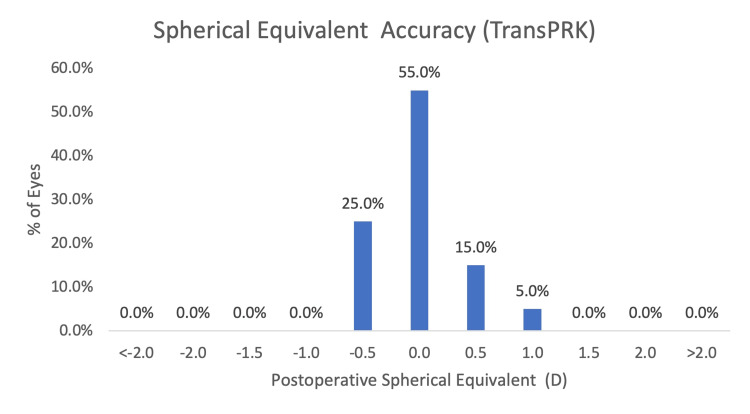
Spherical equivalent accuracy of transepithelial PRK. PRK: Photorefractive keratectomy.

**Figure 6:**
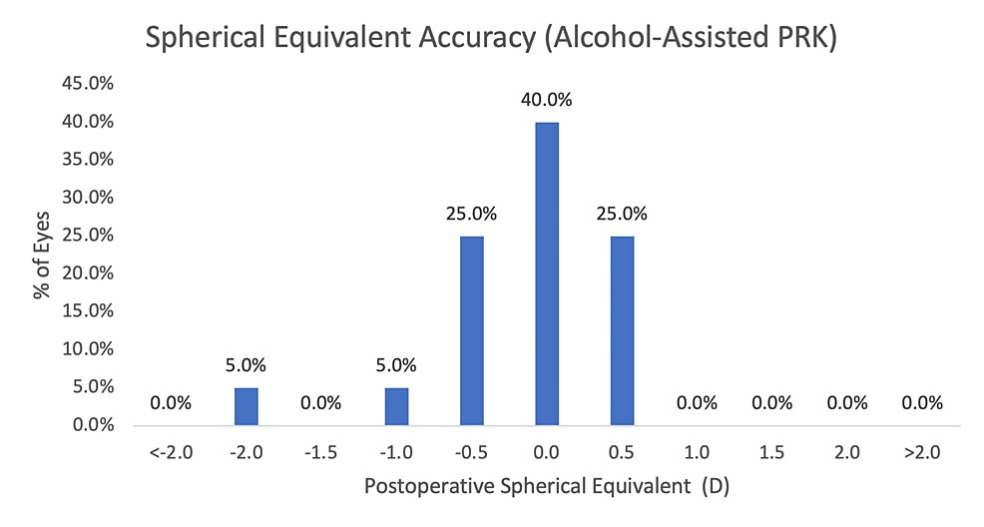
Spherical equivalent accuracy of alcohol-assisted PRK. PRK: Photorefractive keratectomy.

At a mean follow-up of approximately one year after the procedure, eyes in the transepithelial PRK group had a lower amount of residual SE (-0.01 ± 0.32 [-0.50 to 0.88]) than the alcohol-assisted PRK group (-0.09 ± 0.58 [-2.00 to 0.63]). However, the difference was not statistically significant (P = 0.72, Table 3). Also, 95% (n= 19) of the eyes in the transepithelial PRK group and 85% of eyes (n=17) in the alcohol-assisted PRK had residual SE within ±0.5D (P = 0.29). Figures 5-6 show SE accuracy in both groups. The mean amount of corrected SE was -3.54 ± 1.83 (-6.88 to -1.00D) in the transepithelial PRK group and -3.92 ± 1.34D (-6.25 to -1.50D) in the alcohol-assisted PRK group (P = 0.46). The mean spherical correction index was 0.99 ± 0.08 (0.80-1.15) in the transepithelial PRK group and 0.98 ± 0.14 (0.62-1.18) in the alcohol-assisted PRK group (P = 0.71). Regression analysis after controlling for cylinder shows that both groups had similar correction indexes (Rsquare = 0.032, P = 0.75).

Refractive astigmatism (cylinder)

**Figure 7:**
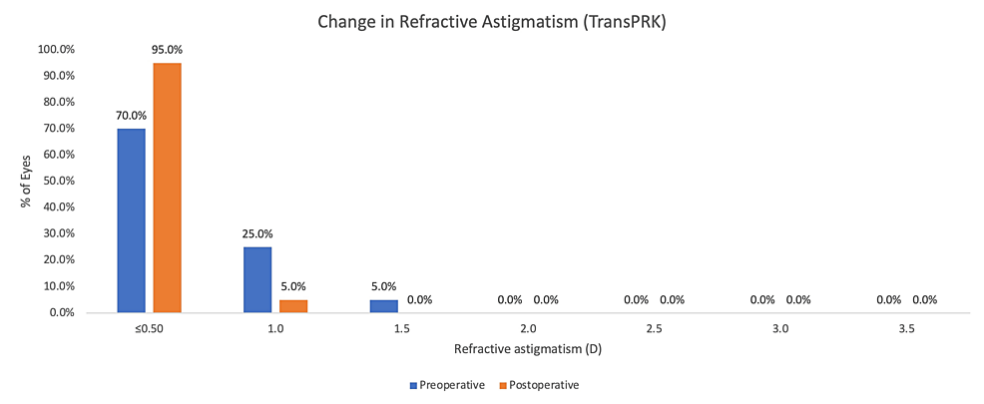
Change in refractive astigmatism of transepithelial PRK. PRK: Photorefractive keratectomy.

**Figure 8:**
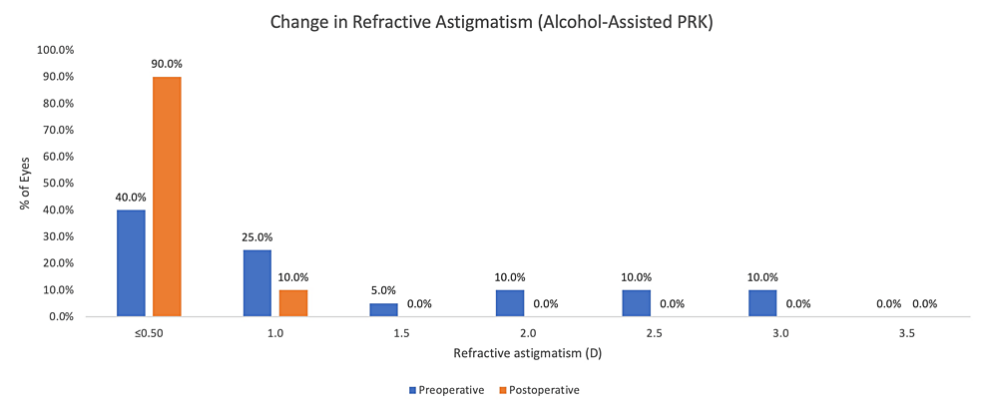
Change in refractive astigmatism of alcohol-assisted PRK. PRK: Photorefractive keratectomy.

At the mean follow-up of one year following the procedure, the mean residual corneal astigmatism was statistically significantly lower in the transepithelial PRK group (0.66 ± 0.51D [0.10 to 2.00D] than the alcohol-assisted group (0.84 ± 0.26D [0.40 to 1.40D]; P = 0.03). However, residual manifest refractive astigmatism was not statistically significantly different between groups (P = 0.87; Table 3). Regression analysis after controlling for cylinder shows that residual astigmatism was not statistically significantly different between groups (Rsquare = 0.394, P = 0.067). No statistically significant difference was noted in the percentages of eyes in either group with cylinders between 0 and 0.50D of refractive astigmatism (P = 0.55). Figures 7-8 show changes in refractive astigmatism in both groups.

Vector analysis

Table 5 summarizes the vector analysis of astigmatic change induced by each surgical procedure. There were no statistically significant differences in difference vector (P = 0.82), surgically induced astigmatism (P = 0.10), and correction index (P = 0.26) between groups. Regression analysis after controlling for cylinder revealed no statistically significant difference in correction indices (Rsquare = 0.172; P = 0.55). The transepithelial PRK group had lower TIA than the alcohol-assisted PRK group (P = 0.01), which corresponds to a lower preoperative cylinder in the transepithelial PRK group than the alcohol-assisted PRK group (P = 0.01, Table 1). The transepithelial PRK group had higher ME (P = 0.05) and AE (P = 0.02) than the alcohol-assisted PRK; Table 4); however, ME was within 0.25D, and AE was <15° for both groups.

**Table 5:** Vector analysis of astigmatic correction. AE: Angle of error; CI: Correction index; D: Diopter; DV: Difference vector; ME: Magnitude of error; PRK: Photorefractive keratectomy; SIA: Surgically induced astigmatism; TIA: Target-induced astigmatism.

	Transepithelial PRK	Alcohol-Assisted PRK	
Parameter	Mean ± SD (range)	Mean ± SD (range)	P-value
DV (D)	0.24 ± 0.26 (0 to 0.76)	0.25 ± 0.33 (0 to 1.01)	0.82
SIA (D)	0.59 ± 0.51 (0 to 2.14)	0.98 ± 0.77 (0 to 2.82)	0.10
TIA (D)	0.41 ± 0.42 (0 to 1.39)	1.02 ± 0.83 (0 to 2.63)	0.01
CI	1.27 ± 45 (0.68 to 2.06)	1.10 ± 0.38 (0.58 to 2.14)	0.26
ME (D)	0.17 ± 0.27 (-0.29 to 0.75)	-0.04 ± 0.31 (-0.93 to 0.50)	0.05
AE (˚)	12 ± 38 (-80 to 90)	-2 ± 6 (-21 to 7)	0.02

## Discussion

This study compared the outcomes of transepithelial PRK versus alcohol-assisted PRK in treating patients with low-to-moderate myopia with or without astigmatism. We noted that each group had similar preoperative and intraoperative parameters. However, the eyes in the transepithelial PRK group consistently had lower values of cylinder, TZ, AZ, and CCT than the alcohol-assisted PRK group. The lower AZ in the transepithelial PRK group could be due to the need for larger than expected AZ in the alcohol-assisted PRK group. We needed a large AZ in the alcohol-assisted group to avoid alcohol decentration, which would cause an inevitable offset ablation, giving us an inaccurate refractive result. Fattah MA et al. [9] and Antonios R et al. [10] conducted similar retrospective comparisons of transepithelial PRK and alcohol-assisted PRK for myopia with SE up to -10D and follow-up durations of over one year (p= 0.9) [10]. Antonios R et al. reported a similar finding to ours. Their transepithelial PRK group had slightly lower AZ than their alcohol-assisted group and a statistically significantly lower TZ in their transepithelial PRK group than their alcohol-assisted group (P = 0.01) [10]. However, Fattah MA et al.'s retrospective study contradicts ours [9]. They reported that AZ was significantly higher in the transepithelial PRK than in the alcohol-assisted PRK (P = 0.01). They explained that this difference was based on the normogram in transepithelial PRK because a higher OZ was needed to ensure minimum post-ablation target OZ. The longer mean operation time of 37 seconds in the transepithelial PRK group than that of the alcohol-assisted PRK corresponds to the ablation time only without calculating the time for epithelium debridement in the alcohol-assisted group. Unfortunately, given that this was a retrospective study, no data are available for debridement time. Our study established that one year after the operation, transepithelial and alcohol-assisted PRK methods had similar mean UDVA, CDVA, percentages of eyes that achieved 20/20, 20/25, and 20/30 UDVA, percentages of eyes that lost two lines of CDVA, and similar efficacy and safety indices. Both groups also had similar residual SE, percentages of eyes within ± 0.5D of target SE, and residual refractive astigmatism. Antonios R et al. and Fattah MA et al. found that both groups exhibited similar postoperative uncorrected VA (UCVA) and percentages of eyes achieving 20/20, 20/25, and 20/30 UCVA [9,10]. No eyes lost one or two lines of CDVA in both group in either study. Also, they both reported similar mean residual manifest refractive SE postoperatively and similar deviation from the targeted SE for both types of treatment. Both studies found that the transepithelial PRK group had lower postoperative astigmatism compared to the alcohol-assisted PRK group. This lower astigmatism value was statistically significant only in Fattah MA et al.'s study (P = 0.02) [9,10]. Fattah MA et al. explained that this lower astigmatism value in the transepithelial PRK group might be due to a centration mismatch in the alcohol-assisted PRK that caused the laser to fall on the epithelium rather than stroma in the peripheral area. In the transepithelial group, these areas are superimposed [9]. In our study, the lower corneal astigmatism noted in the transepithelial PRK group corresponded to preoperative statistically significant lower cylinder values in the transepithelial PRK than the alcohol-assisted PRK group.

Furthermore, a prospective interventional study comparing transepithelial PRK in one eye with alcohol-assisted PRK in the other eye for myopia with astigmatism not more than 3D found no difference in postoperative UCVA at 3, 6, and 12 months after the procedure with equal safety and percentage of eyes that lost one line of VA in each group [19]. Moreover, even with a shorter follow-up duration, the same outcome was reported in two prospective studies comparing transepithelial PRK with alcohol-assisted PRK for moderate [13] and high [15] myopia at three months. These studies' results support our findings; they found no statistically significant difference between the groups in postoperative UCVA, manifest SE, deviation from the target SE, and safety and efficacy [13,15]. On the other hand, Aslanides IM et al.'s prospective trial found that transepithelial PRK was superior to alcohol-assisted PRK in treating myopia within -6.0 D or more and astigmatism <3.5 D at 12 months [5]. They found a statistically significantly better UCVA in the transepithelial PRK group than in the alcohol-assisted PRK group (P = 0.01). No eyes lost two lines of CVA in the transepithelial PRK group, while 14 eyes did so in the alcohol-assisted PRK (P = 0.03). However, the refractive outcomes (postoperative residual SE at one year, percentage of eyes with SE within ±0.5D of the target) were similar in both groups (P = 0.4 and 0.68, respectively). Also, Naderi M et al. conducted a prospective case-control study and found better UCVA, safety, and efficacy indices in their transepithelial PRK group than the alcohol-assisted PRK group to treat mild-to-moderate myopia <6.0D at two months (P = 0.01) [18]. At six months, the UCVA showed no statistically significant difference between the two methods and showed statistically significantly better refractive outcomes (less residual sphere [P = 0.02] and cylinder [P = 0.01]) in the alcohol-assisted group than the transepithelial PRK group. According to Aslanides IM et al., the main determinant of any transepithelial therapy is the likelihood of variations in epithelial thickness and thickness distribution, undermining the accuracy of refractive outcomes [5,10]. Transepithelial PRK has two ablation profiles: epithelial and stromal. In epithelial ablation, the machine ablates the epithelium at a fixed thickness profile based on the normative database for all patients regardless of their actual individual epithelial thickness. If the epithelium of the patient matches this normative data, they will have optimum results. If their epithelium is thinner than the normative, they will have more ablation from the stroma. Conversely, suppose their epithelium is thicker than the normative data. In that case, some of the energy from the stromal ablation profile will be consumed to ablate the epithelium, resulting in smaller than the planned OZ, as the normogram will adjust the OZ according to the amount of intended stromal ablation. The larger the amount of expected stromal ablation, the lesser the amount of OZ adjustment. However, the achieved target refractive ablation in both situations will not be affected [10,13,16]. For the thickness distribution, if the difference in the applied epithelial thickness distribution from the center to the periphery in the ablation profile is larger than the actual difference, a more refractive error will be induced [16]. If the epithelium from the center to the periphery remains of equal thickness, a hyperopic shift by 0.75D will result [16].

Luger MH et al. reported a statistically significant but slight overcorrection in the transepithelial PRK group at three months compared to the alcohol-assisted PRK group (P = 0.04) [16]. They claimed this slight overcorrection was secondary to the difference between the actual and applied epithelial thickness distribution from the center to the periphery. Our study had several limitations. Previous studies reported problems with patients' pain response on questionnaires, categorizing pain intensities and epithelial size and thickness estimations [9,18,19,21]. Given our study's retrospective nature, we could not conduct a similar evaluation of patient responses, and our study generally had the same limitations expected with a retrospective approach. A relatively small sample size limited our study. Also, we did not compare the total duration of surgery between the treatment groups. However, to our knowledge, our study is considered among the first few recent reports that used the Alcon Wave-Light® EX500 machine in the transepithelial type of PRK [24,25].

## Conclusions

In conclusion, for patients with low-to-medium myopia with or without astigmatism, single-step transepithelial PRK yields identical VA, refractive, efficacy, and safety profiles to alcohol-assisted PRK. However, findings reported in the literature are not entirely consistent, and randomized controlled trials with large sample sizes and long-term follow-up are warranted to support our findings further before our conclusions can be broadly applied.
